# ROHHAD Syndrome: Reasons for Diagnostic Difficulties in Obesity

**DOI:** 10.4274/jcrpe.1432

**Published:** 2014-12-05

**Authors:** Pınar Kocaay, Zeynep Şıklar, Emine Çamtosun, Tanıl Kendirli, Merih Berberoğlu

**Affiliations:** 1 Ankara University Faculty of Medicine, Department of Pediatric Endocrinology, Ankara, Turkey; 2 Ankara University Faculty of Medicine, Department of Pediatric Intensive Care, Ankara, Turkey

**Keywords:** ROHHAD syndrome, obesity, hypothalamic dysfunction

## Abstract

A very rare syndrome of rapid-onset obesity with hypoventilation, hypothalamic dysfunction and autonomic dysregulation (ROHHAD) has been recently described as causing morbidity due to hypothalamic dysfunction and respiratory arrest. Its prognosis is poor and often cardiac arrest occurs due to alveolar hypoventilation. This disorder can mimic genetic obesity syndromes and several endocrine disorders. We present a 13-year-old female patient who was reported to be healthy until the age of 3 years. She was admitted to our emergency department, presenting with respiratory distress. Features matching ROHHAD syndrome such as rapid-onset obesity, alveolar hypoventilation, central hypothyroidism, hyperprolactinemia, Raynaud phenomenon and hypothalamic hypernatremia were detected in the patient. In addition to these features, the patient was found to have hypergonadotropic hypogonadism and megaloblastic anemia. Because of its high mortality and morbidity, the possibility of ROHHAD syndrome needs to be considered in all pediatric cases of early- and rapid-onset obesity associated with hypothalamic-pituitary endocrine dysfunction.

## INTRODUCTION

Identified in 78 patients to date, rapid-onset obesity with hypoventilation, hypothalamic dysfunction and autonomic dysregulation (ROHHAD) syndrome is a very rare disease characterized by ROHHAD (1,2,3,4,5,6,7). The disease is now also called ROHHAD-neuroendocrine tumors (ROHHADNET) because it is accompanied by ganglioneuroma located in the abdomen and lungs and NET such as ganglioneuroblastoma in about 40% of the patients (8).

Patients with ROHHAD syndrome usually present at ages between 2 and 4 years with hyperphagia and dramatic weight gain (2,4,7). They are reported to be healthy prior to the appearance of these symptoms. During the subsequent months and years, the following disorders can be encountered: hypothalamic dysfunction, hypernatremia or hyponatremia manifested by thirst and antidiuretic hormone secretion abnormalities, hyperprolactinemia, central hypothyroidism, precocious or delayed puberty, growth hormone (GH) deficiency, adrenocorticotropic hormone (ACTH) deficiency and in parallel autonomic dysfunction, light-nonresponsive pupils, impaired gastro-intestinal motility (constipation), body temperature disorders (hypothermia, hyperthermia), sweating disorders, reduced pain sensation and alveolar hypoventilation (9). Since these cases are rare and there are other disorders that present with obesity, the diagnosis becomes rather difficult.

In this article, we present the complex case of a patient with ROHHAD syndrome whose symptoms developed at different ages and whose diagnosis took a long time since each symptom was evaluated separately at different medical centers.

## CASE REPORT

A thirteen-year-old female patient who presented with respiratory distress and cyanosis was admitted to the emergency department of a different medical center one week ago. The patient’s condition was reported to deteriorate with development of pneumonia and respiratory failure and she was referred to our hospital because of increasing respiratory distress. At admission to our intensive care unit (ICU), the patient was in a drowsy state. The breathing was shallow and she was cyanotic. Respiratory acidosis was determined in the blood gas analysis. She was intubated with assisted ventilation.

The medical history of the patient revealed that her parents were unrelated and healthy, that she was born at term and weighed 3500 grams at birth and that she had two healthy siblings. We were informed that she had started overeating at age 3 years, had gained weight rapidly after that age and had been referred to different medical centers with these complaints. She was reported to have her first menstruation at age 12 years. According to her record at another hospital where she was seen at age 10 years, her body measurements were as follows: body weight: 51 kg, relative body mass index (RBMI) 166%, BMI: 31.62 kg/m2, height: 127 cm and height standard deviation score (SDS): -3.04. She was reported to be obese in appearance and her puberty rating was at Tanner Stage 2. Acanthosis nigricans was identified in the axilla and neck and coldness in hands and feet suggestive of Raynaud phenomenon was noted. The other system findings were found to be normal. These previous records included presence of hypernatremia in the laboratory tests and a diagnosis of diabetes insipidus (DI) despite absence of polyuria and polydipsia. No medication for DI was given, but levothyroxine treatment was started due to primary hypothyroidism. Although cranial magnetic resonance imaging (MRI) findings were reported as normal, bromocriptine treatment was started (dose not recorded) to control hyperprolactinemia. Metformin was started to treat the euglycemic hyperinsulinism noted in the oral glucose tolerance test. The patient was also reported to have cold hands and feet and discoloration, especially increasing in the cold. Presence of Raynaud phenomenon was mentioned also in the follow-up records of different centers. The obese state of the patient could not be brought under control during the follow-up.

Until two years before the patient was admitted to our clinic, her school performance had been satisfactory. Subsequently, a decrease in concentration and decline in school performance were noted. In the past 10 days, these complaints became more evident and drowsiness started.

The patient was admitted to our ICU and was intubated. Her body temperature was 39.2 ˚C, heart rate was 117/min, blood pressure 105/60 mmHg. Her body weight was 69 kg. Height was 145 cm (height SDS -2.28). BMI was calculated as 32.82 kg/m^2^ and RBMI as 159%. The patient had a round plethoric face, buffalo neck, umbilical hernia. Mild acanthosis nigricans in the neck and armpits was present. By Tanner staging, pubertal stage was at stage 4 and other system examinations were normal. 

During the follow-up, the patient’s general condition gradually improved and on the 10th day of hospitalization, she was extubed. After extubation, the patient did not have acidosis, but her PCO2 level continued to remain high. Acute phase reactants were negative, but the feverish state continued. The cause of the fever origin could not be found. Blood and tracheal aspirate cultures were negative. The patient continued to be feverish during her stay in the ICU and after. This fever of unknown cause was thought to be of central origin. The patient’s complete blood count values, renal and hepatic function tests were normal, but hypernatremia was detected (Na: 151 mmol/L). Urine density varied between 1015 and 1017, serum osmolality was normal and no polyuria was observed. During her stay in the ICU, megaloblastic changes were found in her peripheral blood smear and vitamin B12 deficiency was detected.

The patient was referred to our Pediatric Endocrinology Department for reevaluation. Some of the laboratory results are given in Table 1. Serum ACTH (21.01 pg/mL) and cortisol (14.07 µg/dL) levels were normal in the patient, findings which excluded Cushing’s syndrome. Prolactin levels were found to be moderately high. Serum insulin-like growth factor-1 (IGF-1) level was 32.4 ng/mL (-7.92 SD) and IGF binding protein-3 (IGFBP-3) level was 1500 ng/mL (-6.54 SD). These findings and an inadequate response to GH in the GH stimulation test (L-DOPA-induced peak GH response: 0.08 ng/mL) were consistent with GH deficiency. The epiphysis was closed in the left hand-wrist X-ray and the bone age was evaluated as 15 years.

Metformin therapy was discontinued due to normal blood glucose and insulin levels. The patient, who had menarche a year before the application, had secondary amenorrhea. Hypergonadotropic hypogonadism was diagnosed in the laboratory and imaging tests and sex hormone replacement therapy was started. On cranial MRI, cerebral atrophy secondary to the dilatation in second, third and lateral ventricles was detected. The pituitary gland was of normal appearance. Genetic analysis was performed and fluorescence in situ hybridization (FISH) test was negative for Prader-Willi syndrome (PWS).

All these findings indicated the presence of ROHHAD syndrome in our patient. No pathology was found in the abdomen and thorax MRI performed due to the possibility of a NET.

## DISCUSSION

ROHHAD syndrome, which is a rare cause of rapid-onset obesity, may lead to morbidity due to hypothalamic dysfunction and respiratory failure. Its prognosis is poor and often cardiac arrest occurs due to alveolar hypoventilation (10). In this patient, serious episodes of hypoventilation were seen that required assisted ventilation. Therefore, the patient was monitored in the ICU. Alveolar hypoventilation can be associated with different diseases, the most common ones being respiratory and cardiac disorders. In order to mention the existence of central hypoventilation, after ruling out lung and heart diseases, it must also be shown that the patient has no neurological or metabolic diseases (11). In this patient, the chest X-ray was normal and echocardiography showed no cardiac disease. There were no findings of a disease in the metabolic screening. Due to the progression and the clinical findings of the disease, muscle disease was not considered. Genetically, PHOX2B mutation in congenital central hypoventilation syndrome can be a cause of hypoventilation. However, in our patient, the detection of clinically accompanying findings of ROHHAD syndrome enabled us to make a differential diagnosis between these two syndromes (1,7).

Difficulties may be encountered in the diagnosis of ROHHAD syndrome due to the frequency of hyperphagia as a cause of obesity. ROHHAD cases show a normal development up to ages 2 to 4 years, at which time hyperphagia and rapid weight gain are noted. Most findings of ROHHAD syndrome and PWS can overlap (hyperphagia, morbid obesity, hypotonia, mental retardation, short stature, GH deficiency, hypogonadotropic hypogonadism, sleep apnea), thus, PWS must be considered in the differential diagnosis (11). PWS was excluded genetically by the FISH test in our patient.

Leptin-deficient POMC gene mutation, MCR4 gene mutation need also to be considered among the monogenic causes of early-onset obesity (11,12,13). In leptin deficiency, obesity occurs in the early years of life with immunodeficiency symptoms. The patients have red hair. POMC mutations are a distinctive feature of the condition (12). In children affected by mutations in MCR4 gene, as opposed to our case, rapid growth is seen (13). Since there were no distinctive symptoms in the physical and eye examinations, syndromic obesity was not considered in the differential diagnosis of our patient. On the other hand, early-onset facio-truncal obesity with growth retardation, increased urinary free cortisol or nocturnal cortisol are findings indicative of Cushing disease as well, thus, difficulty may be encountered in the differential diagnosis of patients with ROHHAD syndrome who have not yet developed hypothalamic dysfunction or respiratory problems and Cushing disease patients (7). Urinary free cortisol and midnight cortisol levels were found to be normal in our patient.

Hypothalamic dysfunction is a part of ROHHAD syndrome. Hyperprolactinemia, hypothyroidism, adrenal insufficiency, pubertal development abnormalities and unresponsiveness to GH stimulation test may also occur (2,7). Congenital malformations, trauma, hypothalamic tumors and Langerhans cell histiocytosis could all be etiological factors leading to hypothalamic dysfunction in a child. So, radiological imaging of the central nervous system is necessary. The MRI of our patient revealed no findings other than cerebral atrophy.

Central hypothyroidism, hyperprolactinemia, Raynaud phenomenon and electrolyte disorders were all findings which had been identified three years before the patient’s presentation to our clinic, but these findings were not interpreted together to reach a diagnosis.

Our patient had developed secondary amenorrhea. Contrary to our expectations, the laboratory findings were consistent with hypergonadotropic hypogonadism and therefore sex hormone replacement therapy was started.

IGF-1 levels are higher in morbid obesity and these cases are often tall. On the other hand, in patients with ROHHAD syndrome, IGF-1 levels and response to GH stimulatory tests are low (11). Our patient had obesity and short stature. GH tests were consistent with GH deficiency. However, the patient’s bone age had reached 15 years. Because the epiphyses had fused, GH treatment was not considered. The benefit/harm of GH treatment in the hypoventilation or respiratory problems of obese patients is a controversial issue. The presence of IGF-1 receptors in NET may indicate that GH therapy may be risky in such patients (14).

Despite the high serum sodium concentrations in this patient, DI was not considered due to the absence of polyuria and polydipsia. Urine osmolality and urine density were normal.

Body temperature changes, pupillary dysfunction, gastrointestinal dysmotility, decrease in pain sensation can also be observed as types of dysfunction accompanying ROHHAD syndrome. Presence of central fever and Raynaud phenomenon in our patient were suggestive of autonomic dysfunction.

In patients with ROHHAD syndrome, behavioral problems, psychosis, mental retardation, mood and personality changes were defined previously. In our case, there was no mental retardation, but compliance problems and mood changes were reported by the family.

In about 40% of patients, ROHHAD syndrome may be accompanied by neural crest tumor and is called ROHHADNET. Therefore, cases that are suspected of having ROHHAD syndrome should be screened for this possibility as well. No pathological findings were revealed in the abdomen and thorax MRI in our patient.

The gene responsible for ROHHAD syndrome has not been defined as yet. Patwari et al (9) reported monozygotic twins, one of whom was diagnosed to have ROHHAD syndrome, while the other twin was not affected. This finding suggests that autoimmune or epigenetic factors have a role in the etiopathogenesis of the syndrome.

In our patient, interestingly, megaloblastic anemia was found. This association has not been previously reported. This association of megaloblastic anemia with ROHHAD syndrome can be a coincidence or a part of the syndrome. Future publications may contribute to the explanation of these possibilities.

In conclusion, rapid-onset obesity, presence of endocrinological disorders, hypothalamic dysfunction and alveolar hypoventilation in a patient should lead to a consideration of ROHHAD syndrome in the diagnosis. However, different findings starting at different time intervals may create difficulties in establishing the diagnosis.

## Figures and Tables

**Table 1:**
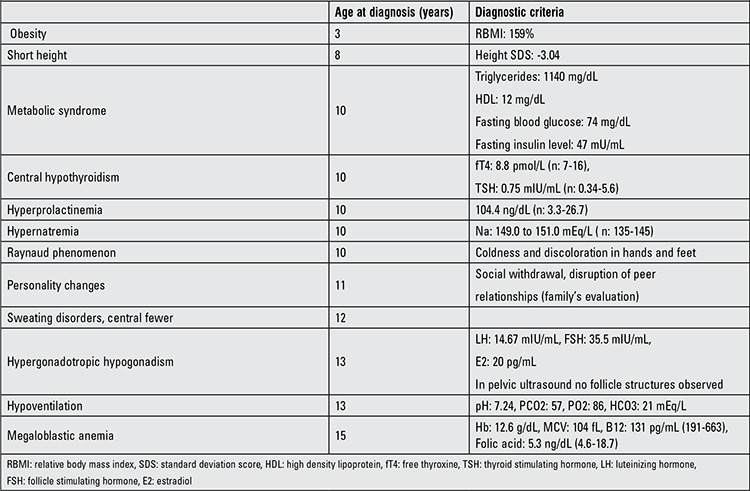
Clinical and laboratory findings of the patient

## References

[1] Katz ES, McGrath S, Marcus CL (2000). Late-onset central hypoventilation with hypothalamic dysfunction: a distinct clinical syndrome. Pediatr Pulmonol.

[2] Ize-Ludlow D, Gray JA, Sperling MA, Berry-Kravis EM, Milunsky JM, Farooqi IS, Rand CM, Weese-Mayer DE (2007). Rapid-onset obesity with hypothalamic dysfunction, hypoventilation, and autonomic dysregulation presenting in childhood. Pediatrics.

[3] Rand CM, Patwari PP, Rodikova EA, Zhou L, Berry-Kravis EM, Wilson RJ, Bech-Hansen T, Weese-Mayer DE (2011). Rapid-onset obesity with hypothalamic dysfunction, hypoventilation, and autonomic dysregulation: analysis of hypothalamic and autonomic candidate genes. Pediatr Res.

[4] De Pontual L, Trochet D, Caillat-Zucman S, Abou Shenab OA, Bougneres P, Crow Y, Cunningham S, Esteva B, Heberle LC, Leger J, Pinto G, Polak M, Shafik MH, Straus C, Trang H, Munnich A, Lyonnet S, Desguerre I, Amiel J (2008). Delineation of late onset hypoventilation associated with hypothalamic dysfunction syndrome. Pediatr Res.

[5] Onal H, Ersen A (2010). A case of late-onset central hypoventilation syndrome with hypothalamic dysfunction: through a new phenotype. Turk J Pediatr.

[6] Paz-Priel I, Cooke DW, Chen AR (2011). Cyclophosphamide for rapid-onset obesity, hypothalamic dysfunction, hypoventilation, and autonomic dysregulation syndrome. J Pediatr.

[7] Bougnères P, Pantalone L, Linglart A, Rothenbühler A, Le Stunff C (2008). Endocrine manifestations of the rapid-onset obesity with hypoventilation, hypothalamic, autonomic dysregulation, and neural tumor syndrome in childhood. J Clin Endocrinol Metab.

[8] Sirvent N, Bérard E, Chastagner P, Feillet F, Wagner K, Sommelet D (2003). Hypothalamic dysfunction associated with neuroblastoma: evidence for a new Paraneoplastic syndrome? Med Pediatr Oncol 2003;40:326-328. 2003;40:326-328..

[9] Patwari PP, Rand CM, Berry-Kravis EM, Ize-Ludlow D, Weese-Mayer DE (2011). Monozygotic twins discordant for ROHHAD phenotype. Pediatrics.

[10] Chew HB, Ngu LH, Keng WT (2011). Rapid-onset obesity with hypothalamic dysfunction, hypoventilation and autonomic dysregulation (ROHHAD): a case with additional features and review of the literature. BMJ Case Rep.

[11] Abaci A, Catli G, Bayram E, Koroglu T, Olgun HN, Mutafoglu K, Hiz AS, Cakmakci H, Bober E (2013). A case of rapid-onset obesity with hypothalamic dysfunction, hypoventilation, autonomic dysregulation, and neural crest tumor: ROHHADNET syndrome. Endocr Pract.

[12] Farooqi IS, O’Rahilly S (2005). Monogenic obesity in humans. Annu Rev Med.

[13] Govaerts C, Srinivasan S, Shapiro A, Zhang S, Picard F, Clement K, Lubrano-Berthelier C, Vaisse C (2005). Obesity-associated mutations in the melanocortin 4 receptor provide novel insights into its function. Peptides.

[14] Tanno B, Negroni A, Vitali R, Pirozzoli MC, Cesi V, Mancini C, Calabretta B, Raschellà G (2002). Expression of insulin-like growth factor-binding protein 5 in neuroblastoma cells is regulated at the transcriptional level by c-Myb and B-Myb via direct and indirect mechanisms. J Biol Chem.

